# An integrative Raman microscopy-based workflow for rapid in situ analysis of microalgal lipid bodies

**DOI:** 10.1186/s13068-015-0349-1

**Published:** 2015-10-06

**Authors:** Sudhir Kumar Sharma, David R. Nelson, Rasha Abdrabu, Basel Khraiwesh, Kenan Jijakli, Marc Arnoux, Matthew J. O’Connor, Tayebeh Bahmani, Hong Cai, Sachin Khapli, Ramesh Jagannathan, Kourosh Salehi-Ashtiani

**Affiliations:** Division of Engineering, New York University Abu Dhabi, P. O. Box 129188, Abu Dhabi, UAE; Laboratory of Algal, Systems, and Synthetic Biology, Division of Science and Math, New York University Abu Dhabi, P.O. Box 129188, Abu Dhabi, UAE; Center for Genomics and Systems Biology (CGSB), New York University Abu Dhabi, P.O. Box 129188, Abu Dhabi, UAE; Core Technology Platform, New York University Abu Dhabi, P.O. Box 129188, Abu Dhabi, UAE

**Keywords:** Algae, Biofuel, Single cell analysis, FACS, Confocal Raman microscopy, Lipidomics

## Abstract

**Background:**

Oils and bioproducts extracted from cultivated algae can be used as sustainable feedstock for fuels, nutritional supplements, and other bio-based products. Discovery and isolation of new algal species and their subsequent optimization are needed to achieve economical feasibility for industrial applications. Here we describe and validate a workflow for in situ analysis of algal lipids through confocal Raman microscopy. We demonstrate its effectiveness to characterize lipid content of algal strains isolated from the environment as well as algal cells screened for increased lipid accumulation through UV mutagenesis combined with Fluorescence Activated Cell Sorting (FACS).

**Results:**

To establish and validate our workflow, we refined an existing Raman platform to obtain better discrimination in chain length and saturation of lipids through ratiometric analyses of mixed fatty acid lipid standards. Raman experiments were performed using two different excitation lasers (*λ* = 532 and 785 nm), with close agreement observed between values obtained using each laser. Liquid chromatography coupled with mass spectrometry (LC–MS) experiments validated the obtained Raman spectroscopic results. To demonstrate the utility and effectiveness of the improved Raman platform, we carried out bioprospecting for algal species from soil and marine environments in both temperate and subtropical geographies to obtain algal isolates from varied environments. Further, we carried out two rounds of mutagenesis screens on the green algal model species, *Chlamydomonas reinhardtii*, to obtain cells with increased lipid content. Analyses on both environmental isolates and screened cells were conducted which determined their respective lipids. Different saturation states among the isolates as well as the screened *C. reinhardtii* strains were observed. The latter indicated the presence of cell-to cell variations among cells grown under identical condition. In contrast, non-mutagenized *C. reinhardtii* cells showed no significant heterogeneity in lipid content.

**Conclusions:**

We demonstrate the utility of confocal Raman microscopy for lipid analysis on novel aquatic and soil microalgal isolates and for characterization of lipid-expressing cells obtained in a mutagenesis screen. Raman microscopy enables quantitative determination of the unsaturation level and chain lengths of microalgal lipids, which are key parameters in selection and engineering of microalgae for optimal production of biofuels.

**Electronic supplementary material:**

The online version of this article (doi:10.1186/s13068-015-0349-1) contains supplementary material, which is available to authorized users.

## Background

New algal isolates displaying interesting lipid phenotypes have been targets of biofuel production efforts. For example, a recently isolated cold-tolerant lipid producing green–yellow algae, *Heterococcus* sp. DN1 accumulates lipids to up to 55 % of its dry weight and produces eicosapentaenoic acid (EPA) at near freezing temperatures [1]. A high-lipid strain of *Scenedesmus* sp. strain R-16, which accumulates lipids at 43.4 % of its dry weight, was selected for heterotrophic lipid production by screening a group of 88 field isolates using ultrasonic assisted Nile red lipid staining [2].

Following strain isolation, mutant selection and engineering can increase lipid production. The lipid output, which can be as high as 80 % of the dry mass of the cell [3], often depends on various nutritional stresses such as nitrogen or phosphorous starvation [4] which should be investigated and optimized. Additionally, metabolic engineering and genetic modification can lead to the maximization of lipid or other metabolite production. Genetic knockouts, transformations or UV mutagenesis can alter the expression of lipids or other metabolites of interest. Because of the ease of UV mutagenesis, it is an attractive and quick initial genetic modification technique that can be applied on different algal isolates to perturb their lipid production. For example, Vigeolas et al. [5] used UV mutagenesis to increase the lipid production of *Chlorella sorokiniana* and *Scenedesmus obliquus* isolates. Following UV mutagenesis, they used Nile red dye to screen for increased lipid production among the mutants and showed that certain mutants had an obvious increase in lipid production without a noticeable change in growth rate [5].

One automated lipid screening approach is through sorting mutants based on lipid production using fluorescence activated cell sorting (FACS). FACS allows for automatic sorting on a single cell based on the fluorescence of a dyed cell. It is a non-invasive and non-lethal method that allows great flexibility in sorting. In FACS, the fluorescence signal of heterogeneous cell mixtures is read one cell at a time, and that signal determines the charge that will be induced onto the cell. The charged cells are then deflected into separate wells, ultimately resulting in sorting based on a fluorescence characteristic. Besides fluorescence, cells can be sorted based on size or complexity or a combination of size, complexity and fluorescence parameters.

Given a genetic or metabolic change to algal lipid production, technological advancements will require the development of methods to precisely identify and quantify the lipids generated by microalgae and to correlate the generated lipids to the various genetic manipulation strategies and/or growth conditions [6, 7]. Availability of such techniques would enable selection of microalgae necessary for the optimal production of biofuels based on chemical characteristics in addition to lipid production quantities. Analytical techniques used to investigate lipids in algal research include GCMS [8], LCMS [9], NMR [10], FTIR [11], and Raman spectroscopy [12]. Raman spectroscopy is advantageous in that it allows the label-free, rapid characterization of biological cells [7, 12]. Unlike most other methods of lipidomics, it does not require extraction of lipids from the cells and can be applied for in vivo analyses [13]. In Raman spectroscopy, high intensity monochromatic radiation, usually from a laser, is shined on a sample and the scattered radiation is analyzed in terms of the energies (frequencies) of the scattered photons. Micro-Raman spectroscopy yields information on a single cell level and is useful for studying cellular dynamics. The scope of Raman spectroscopy is further expanded by advances such as surface enhanced Raman spectroscopy (SERS) [14, 15], coherent anti-stokes Raman scattering (CARS) [16, 17], resonance Raman spectroscopy (RRS) [18, 19] and confocal Raman microscopy (CRM) [20, 21]. CARS is a non-linear technique that overcomes the problem of Raman effect being a non-resonant phenomena. It allows for a much faster 3-D imaging but is limited by the spectral bandwidth available. Confocal Raman spectroscopy on the other hand, allows access to full spectral information with high spatial resolution. Combination of confocal optical microscopy with Raman spectroscopy resulting in 3-D spatial characterization of the samples has resulted in Confocal Raman microscopy becoming the method of choice for label-free and real-time monitoring of various biological samples and living cells investigations [12, 22]. Using direct or serial imaging techniques (e.g., point and line mapping) confocal Raman microscopy enables fast spectral data acquisition, at a reasonable spatial resolution of ~300 nm.

In algal research, Raman spectroscopy has been used for the analysis of pigments, carbohydrates, and lipids [23, 24]. Huang et al. [25] used confocal Raman microscopy for the compositional analysis of microalgae. The investigators collected Raman spectra using 532 nm laser and found strong fluorescence background with time-dependent behavior. Raman spectroscopy has also been used in single cell resolution analysis of microalgae. The prediction of nutrient status of single microalgal cells by Raman spectroscopy is reported by Heraud et al. [26]. Kaczor et al. [27] have reported imaging of astaxanthin in a single microalgal cell by in situ Raman imaging with 1064 nm laser. Recently, Wu et al. [7] developed a laser trapping Raman spectroscopy (LTRS) method, a combination of laser trapping and micro-Raman spectroscopy, to analyze the lipid composition in microalgae on a single cell basis. In addition, Hosokawa et al. [28] used the confocal Raman microscopy technique for quantitative monitoring of lipids on a single cell basis. A concise summary of the developments in Raman spectroscopy based algal research is provided in two recently published review articles [29, 30]. Accordingly, the ratiometric method of lipid analysis has been established with a sound footing, though fine-tuning of acquisition parameters is necessary for improved data collection and more accurate analysis.

## Results and discussion

### Experimental workflow

**Fig. 1:**
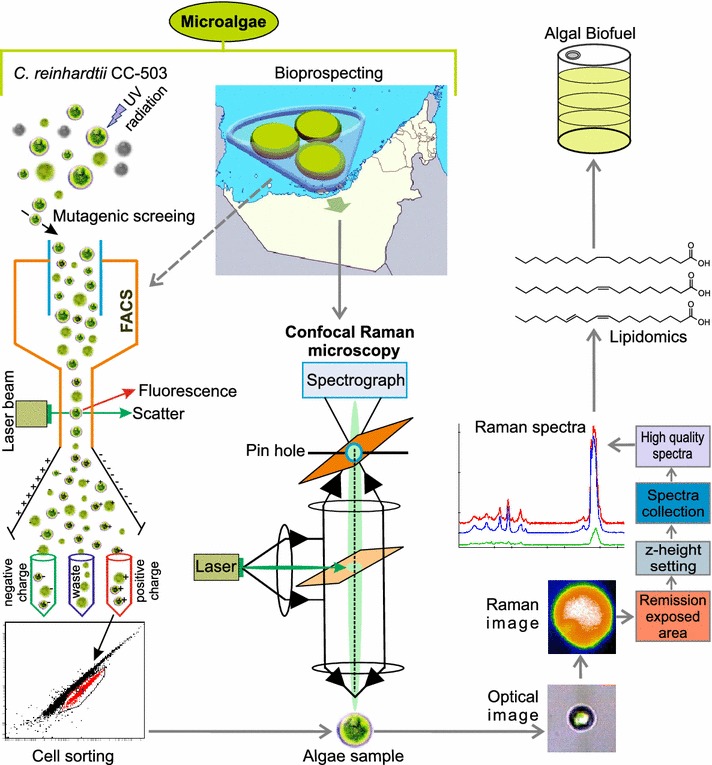
The workflow employed in lipid characterization of microalgae. Bioprospecting of aquatic and soil algae, as well as mutagenesis of algal cells, such as *C. reinhardtii*, are carried out to generate algal samples with potentially desirable lipid characteristics. Mutagenized cells are sorted by FACS, based on fluorescence of a lipophilic dye to isolate cells with increased lipid accumulation phenotypes. The obtained environmental samples and screened mutants are then analyzed by confocal Raman microscopy. This method, once optimized, allows for rapid in situ characterization of lipids through ratiometric analysis of Raman spectra. As a rapid process, this workflow offers better spatial resolution and characterization of about 10 cells per hour. The spectra yield information about the number of C=C bonds and the hydrocarbon chain length of the lipid molecules. The workflow allows rapid characterization of algae for molecular traits that are suitable for use in production of biofuels

Bioprospecting and mutagenesis are two important strategies in algal-based biofuel development. Optimization of biofuel production using these strategies requires the sorting and analysis of a large number of algal isolates in terms of their triacylglycerol (TAG) contents. FACS and ratiometric Raman analysis are ideally suited for this purpose and we demonstrate the application of both in an integrated workflow for screening of microalgae after bioprospecting and UV mutagenesis. Isolation of novel algae from field samples may be followed by UV mutagenesis to perturb and improve lipid production. Following UV mutagenesis, FACS can select for mutant populations and strains that have a change in lipid production. Central to this workflow, confocal Raman microscopy can characterize lipids produced by the sorted and selected algal cells in situ rapidly without the need to extract lipids from the cells (Fig. 1). Optical micrographs using 50× objective are recorded to locate single cells and forwarded for a controlled photo-bleach at *λ* = 532 nm (excitation). The approximate locations of lipid-rich regions are identified by Raman hyperspectral imaging with low pixel density (10 × 10 spectra/image at 0.5 s integration time). These parameters enable a faster acquisition of Raman hyper spectral images. The selection of suitable filters helps to locate high lipid concentration regions. The size of the lipid-rich regions is reduced until the scanning area ~(1 × 1) µm^2^ is obtained. Single spectrum is extracted at highest integrated peak location by z-focus tuning. As a rapid process, this workflow offers better spatial resolution and characterization of around 10 cells per hour (Additional file 1). To validate this workflow, field isolation is demonstrated on six isolated strains, while UV mutagenesis and FACS screening is demonstrated on *C. reinhardtii* (CC-503). Confocal Raman microscopy is then applied to samples to characterize the lipid contents.

### Raman spectroscopy of algae

Essential to any algal lipid bioproduction endeavor is the ability to rapidly characterize accumulating algal lipids. Confocal Raman microscopy can fulfill this role on a single cell and high throughput basis. Hyperspectral imaging using confocal Raman microscopy was used to locate lipid-rich regions within microalgae cells with a high spatial resolution. Integrated peak intensity corresponding to the most intense Raman band (2800–3000 rel cm^−1^ when using the green laser for excitation and 1440 rel cm^−1^ when using the near infra-red {NIR} laser for excitation) used as a signature peak to locate lipid-rich regions within the cells (Additional file 1). We note that the ratiometric method of analysis is applicable for the characterization of free fatty acids as well as triacylglycerides (TAGs) and that the accumulated algal lipids are expected to be in the form of TAGs [31]. When used for the analysis of TAGs, it provides an estimate of the average number of C=C bonds per fatty acid residue and the average chain length of the fatty acid residues incorporated in the TAGs. We used two different excitation lasers, 532 and 785 nm, with a close agreement observed between the two measurements. These two lasers differ in terms of the excitation of the fluorophores present in microalgae as well as their cross sections for the excitation of lipid Raman bands. Laser-induced autofluorescence is strong with the 532 nm laser but the Raman excitation power of the laser is ~5 times higher than that of the 785 nm laser. For the 532 nm laser excitation, optimized spectral conditions for photobleaching of algal cells were developed to obtain fluorescence-free Raman spectra. Spectral data acquisition and imaging were tuned for optimal integration time and number of accumulations, resulting in significantly improved lipids signal to noise ratios. Calibration of quantitation using fatty acids standards and ratiometric analysis of Raman spectra obtained through this technique to determine the number of C=C bonds and the hydrocarbon chain length of the lipid molecules are described in the following sections.

### Raman spectra of fatty acid standards

**Fig. 2:**
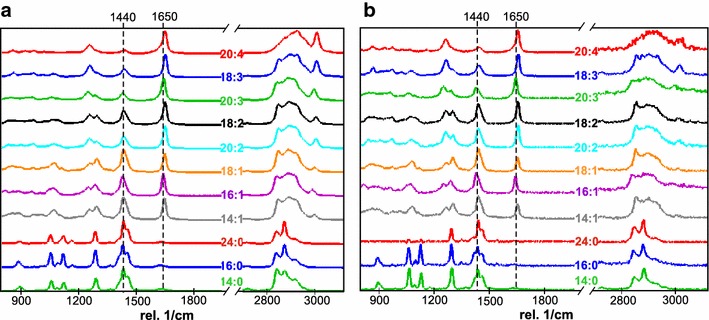
Raman spectra of fatty acid standards and microalgal lipids **a** green excitation (532 nm laser) and **b** NIR excitation (785 nm laser). Raman spectra are listed with decreasing degree of unsaturation of fatty acid standards from *top* to *bottom* and show a gradual decrease in the intensity of Raman band at 1650 rel cm^−1^ (C=C stretching mode) compared to that of 1440 rel cm^−1^ band (CH_2_ bending mode). Raman peaks in the 2800–3000 rel cm^−1^ (CH_2_ symmetric and asymmetric stretches) are scaled in intensity to allow visualization. These modes are more intense than the peaks at 1440 and 1650 rel cm^−1^ when using the green excitation and much weaker than the peaks at 1440 and 1650 rel cm^−1^ when using the NIR excitation

We selected a series of eleven pure fatty acids as calibration standards for the ratiometric method of determination of lipid composition. These even-numbered fatty acids are commonly found in micro-algal lipid extracts and are characterized by differences in their aliphatic chain lengths and the number of C=C bonds (Additional file 2). Figure 2, shows the Raman spectra of these standards with decreasing degrees of unsaturation. The dominant peaks for the 532 nm excitation (Fig. 2a) are due to –CH_2_ symmetric and asymmetric stretching vibrations, centered at 2800–3000 cm^−1^, whereas the dominant peaks for the 785 nm excitation (Fig. 2b) are the –CH_2_ bending peaks at 1440 cm^−1^. The absolute peak intensities of the dominant peaks for spectra obtained with 532 nm excitation are roughly an order of magnitude higher than those obtained using the 785 nm excitation. Peaks at 1260 cm^−1^ (=C–H *cis* stretch), 1650 cm^−1^(C=C stretch), and 3023 cm^−1^ (=C–H stretch) are the markers of unsaturation whereas peaks at 1300 cm^−1^ (–CH_2_ twist), 1440 cm^−1^ (–CH_2_ bend), and ~2800–3000 cm^−1^ (CH_2_ symmetric and asymmetric stretches) correspond to the aliphatic chains of fatty acids [7].

Raman spectra for *C. reinhardtii* (CC-503) were recorded for 532 and 785 nm excitation wavelengths (Additional file 3) after background correction. These data describe Lorentzian peak curve fitting for the oleic acid and the reference microalgae *C. reinhardtii* (CC-503) in the lipid-rich region at 1445 and 1650 cm^−1^ and evaluation of their intensity ratios *I*_1650*/*_*I*_1440_. The Raman band at 1650 cm^−1^ could overlap with the amide (*I*) band from proteins. In these measurements, we have made sure that the spectra selected for analysis were free from other characteristic protein bands such as δ(N–H) and ν(N–C) bands (amide (III) bands) at 1220–1300 cm^−1^, disulfide peaks at 550 cm^−1^ and characteristics peaks of aromatic amino acids near 1004 cm^−1^ [7]. Moreover, Lorentzian peaks curve fitting were performed for olefinic (=C–H stretch) band at 3003 cm^−1^ that appears immediately after –CH_2_ stretch ~2800 cm^−1^. The curve fitting data for this characterization are included in Additional file 3. A comparison of the intensity ratios *I*_1650_/*I*_1440_ and *I*_3003_/*I*_1440_, is found to be satisfactory as both intensity ratios, *I*_1650_/*I*_1440_ and *I*_3003_/*I*_1440_, corresponding to CC-503 (row 3) are close to the values obtained for the same fatty acid standard (oleic acid, row 2). Thus, both methods of analysis are in good agreement with respect to number of C=C bonds and CH_2_:C=C ratio of the algal lipids.

In addition to the lipid bands mentioned before, peaks in the 1400–1500 cm^−1^ spectral range were seen due to C–C stretching in chlorophyll a, as well as CH_2_ and CH_3_ deformation modes from β-carotene and chlorophyll a. The bands 1000–1200 cm^−1^ are due to C–O stretching vibrations of chlorophyll and sharp bands at 1155 and 1006 cm^−1^ could be indexed to C–H stretching in β-carotene. NCC and CCC in-plane bending of chlorophyll appeared around 800–1000 cm^−1^, whereas OCO and CH deformation are found below 800 cm^−1^. Excitation with 785 nm laser resulted in enhanced β-carotene bands. Increase in β-carotene bands due to NIR excitation is usually associated with π-electron/phonon coupling mechanism consistent with previous reports [32, 33].

### Quantification of standards and microalgal lipids

**Fig. 3:**
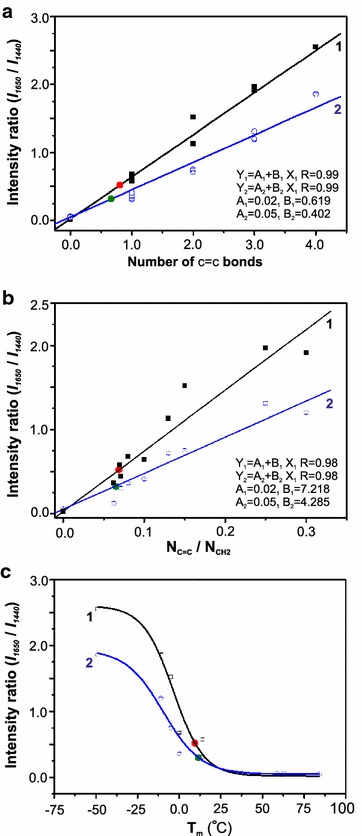
Calibration curves for quantitative assessment of extracted micro-algal lipids. Integrated peak intensities obtained from Raman spectra of fatty acid standards were used to calculate the intensity ratio *I*_1650_/*I*_1440_. **a** shows the linear variation of intensity ratio with the degree of unsaturation (i.e., C=C bonds), **b** shows the linear variation of intensity ratio with N_C=C_/N_CH2_ (i.e., ratio of the number of C=C bonds with the number of –CH_2_– units), and **c** shows the sigmoidal variation of intensity ratio with melting points of fatty acid standards. *Black line* *(1)* represents curve-fit to the data obtained using 532 nm excitation and *blue line* *(2)* represents curve-fit to the data obtained using 785 nm excitation. Results of the ratiometric analysis of lipids expressed in *C. reinhardtii* (CC-503) using the two lasers are shown by *red* and *green circles* on the respective calibration curves

Ratiometric analysis was used for quantitative analysis of lipids since absolute spectral intensities with low variation are not easily obtainable in Raman spectroscopy. This is especially true for complex samples such as lipids in cellular environments because of the background from fluorescence and other optical phenomena. Specifically, the ratios of integrated peak intensities at 1440 and 1650 cm^−1^, (*I*_1650_/*I*_1440_), corresponding to the –CH_2_ bending and C=C stretch, respectively, in the lipid-dominated region were used for compositional analysis. For lipids, the ratio of *I*_1650_/*I*_1440_ has been shown to be linearly correlated with both the number of C=C bonds and N_C=C_/N_CH2_, where N_CH2_ is the number of aliphatic –CH_2_– groups [7]. Figure 3a, b show the calibration plots for intensity ratios of *I*_1650_/*I*_1440_ recorded using 532 and 785 nm laser with number of C=C bonds and N_C=C_/N_CH2_ in standard lipids. Increasing the degree of unsaturation led to a linear increase in intensity ratio, which is in agreement with Wu et al. [7]. The ratio of *I*_1650_/*I*_1440_ also exhibits a non-linear correlation with the melting points of lipids (Fig. 3c). Calibration plots for even-numbered fatty acid standards with the integer values for the degree of unsaturation are linear, but in our current investigation, the intensity ratios calculated for micro-algal lipids from these plots are non-integers. The non-integer values suggest the presence of a mixture of fatty acids with different chain lengths and degree of unsaturation in the algal samples. In order to obtain the exact proportion of fatty acids and intermediate values of C=C or N_C=C_/N_CH2_ in the calibration plot, we needed to obtain Raman spectra for mixtures of these fatty acids. For example, two fatty acids with similar physical characteristics could be mixed in different weight fractions. The idea of using mixtures of standard lipids can lead to improved accuracy of the data analysis. Hence, we mixed two standard fatty acids, oleic and palmitoleic acid, in different proportions and intermediate values of intensity ratios were obtained and included in the calibration plot (Additional file 4), the results thus obtained with mixed standard lipids are consistent with the unmixed calibration plots.

### Verification and analysis of *C. reinhardtii* strain CC-503 lipid content

Single cell Raman spectra were collected from *C. reinhardtii*, the reference strain (CC-503) microalgal species using 532 and 785 nm lasers. Prior to spectral acquisition, the cells were photobleached using optimized laser power values (see “Methods”), to minimize the autofluorescence from the sample area. Peak assignment information for the recorded spectra can be found in Additional file 5. Before photobleaching, the spectrum was dominated by the fluorescence of pigments as well as the resonance Raman peaks of carotenoids. Carotenoids are lipid soluble and can give rise to intense resonance Raman peaks even at low concentrations. The carotenoid peaks are present near the unsaturation and saturation marker locations (Additional file 5) and could introduce error in measurement. Therefore, controlled photobleaching was performed until the intensities of the most dominant carotenoid peak (1520 cm^−1^) decreased to levels far below the lipid analysis peaks (1440 and 1650 cm^−1^). After photobleaching, Raman hyperspectral imaging was performed to locate an area with the maximum lipid contribution to Raman spectra, as described in “Methods” (see also Additional file 1). The analysis volume contains mostly lipid-dominated regions in the confocal focus and is characterized by a Raman spectrum that is almost similar to that of the standard lipids. Thus, our guided scan locates lipid droplets with high precision and results in better quality spectra.

Calibration plots were obtained with visible and NIR laser excitations along with the analysis of *C. reinhardtii* (CC-503) lipids as a reference microalgae (Fig. 3). The intensity ratio (*I*_1650_/*I*_1440_*)* calculated for 532 and 785 nm excitation were found to be 0.52 and 0.30, respectively. In Fig. 3a, the corresponding number of C=C bonds are found to be 0.80 and 0.66, respectively. The ratio of C=C bonds with –CH_2_ stretching (N_C=C_/N_CH2_) (Fig. 3b) are found to be 0.07 and 0.06, respectively. Therefore, the number of C=C and N_C=C_/N_CH2_ calculated by 532 and 785 nm laser are in good agreement with each other. This result demonstrates that rigorous, quantitative comparison of spectral data can be obtained regardless of the excitation source. For this well-characterized species, i.e., *C. reinhardtii*, our measured values are consistent with those reported in the literature which report the occurrence of saturated and monosaturated lipids predominantly, with a variation in the relative concentration depending on the growth conditions [34–37]. Additionally, the melting point for the algal sample, *C. reinhardtii,* CC-503, obtained from the sigmoidal plot, was also quantitatively consistent with the results obtained for the standard fatty acids (Fig. 3c). The melting point of this lipid was found to be 10 (±2) °C. We performed liquid chromatography–mass analysis (LC–MS) for independent validation of the Raman analysis. As shown in the supplementary information section (Additional file 6) the major component of the extract is oleic acid, which confirms the results of ratiometric Raman analysis.

### Isolation of novel aquatic and soil microalgal strains

**Fig. 4:**
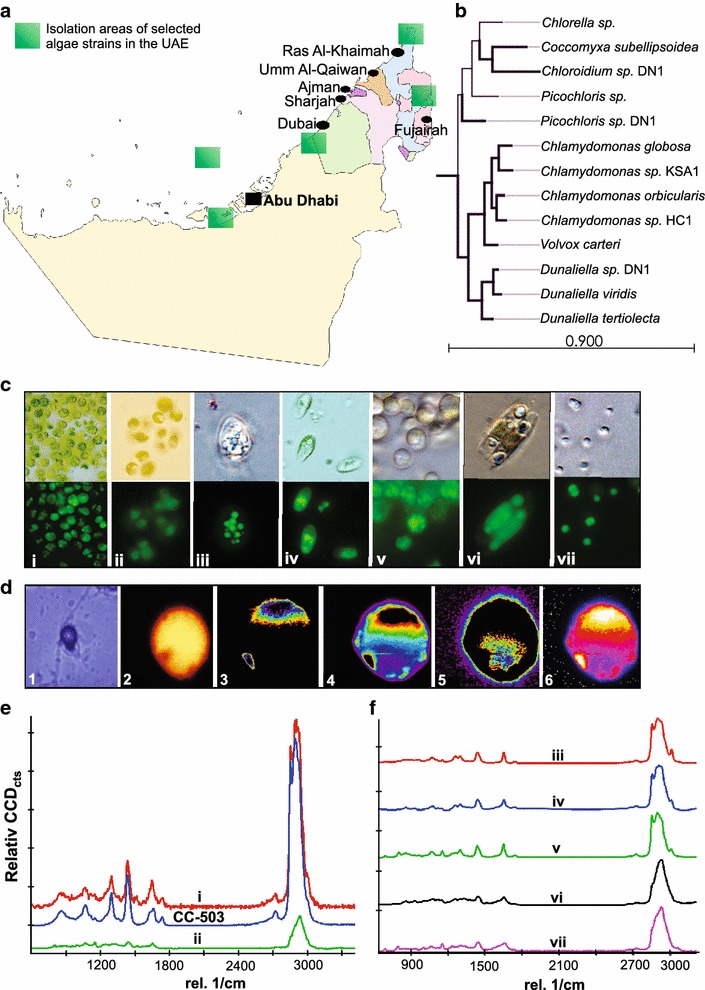
Bioprospecting and characterization of isolated algal strains. **a** Explored areas for isolation of selected algae strains in the UAE are shown in the map (Map of the UAE is adapted and modified from Google Maps). **b** Phylogenetic tree of novel algal isolates. The tree was reconstructed using a 1400 nt region of the RbcL gene from novel algal isolates and NCBI-cataloged species are shown. Maximum likelihood phylogenies are inferred. Evolutionary distances were measured using a Jukes-Cantor method with 1000 bootstrap replicates. **c** Brightfield and fluorescence micrographs of algal isolates, (*i*) *Chlamydomonas* sp. KSA1, (*ii*) *Chlamydomonas* sp. HC1, (*iii*) *Chloroidium* sp. DN1, (*iv*) *Dunaliella* sp. DN1, (*v*) MG8 (unknown lineage), (*vi*) RSSF (unknown lineage) and (*vii*) *Picochloris* sp. DN. Fluorescence micrographs are dyed with *Nile red* or BODIPY 505/515 to highlight lipid bodies. **d** Optical micrograph (*1*) and Raman hyperspectral image (*2*), of reference *C. reinhardtii* (CC-503) microalgae and constructed Raman images of proteins (1003 cm^−1^) (*3*), carotenoid components (1520 cm^−1^) (*4*), lipid bodies (1445 cm^−1^) (*5*), and combination of these components (*6*). **e** Raman single spectrum collected for *C. reinhardtii* (CC-503) strain and isolated soil microalgae and **f** isolated aquatic microalgae. Spectra were recorded using 532 nm laser as excitation source after performing controlled photobleaching to reduce the fluorescence background; the algal strain designations are the same as in *panel* **c**

To demonstrate the applicability of our workflow to a range of novel algae that might be isolated through bioprospecting expeditions, we isolated algae from a number of different environments including temperate and subtropical geographies (Fig. 4a). Genomic DNA was extracted and sequenced from most of the isolates. Following a draft de novo assembly, phylogenetic assignments were carried out based on sequence alignment of the Ribulose-1,5-bisphosphate carboxylase/oxygenase (RuBisCo) large subunit, RbcL (Fig. 4b, “Methods”).

Two novel species of Chlamydomonas were isolated from soil samples from New York City, USA (*Chlamydomonas* sp. KSA1) and the outskirts of Abu Dhabi, UAE (*Chlamydomonas* sp. HC1). The isolated Chlamydomonas strains were more similar (with regards to nucleotide sequence of key taxonomic regions) to *Chlamydomonas reinhardtii* than the majority of other Chlamydomonas strains except for *Chlamydomonas orbicularis* and *Chlamydomonas globosa* (Fig. 4b). Five strains were isolated from aquatic samples: two strains were isolated from coastal seawater samples of the UAE and San Francisco bay area (USA), one strain was isolated from the southwestern desert of Abu Dhabi (UAE), one strain was isolated from a saline mangrove sample from the Abu Dhabi Mangroves, and one strain was isolated from fountain water on the Abu Dhabi Corniche (Fig. 4a). Of the four UAE-isolated strains, *Dunaliella* sp. DN1 was isolated from hot desert mud after a rare rainfall in the region, *Picochloris* sp. DN1 was isolated in coastal Arabian Gulf waters, and *Chloroidium* sp. DN1 was isolated from municipal water in downtown Abu Dhabi (Fig. 4a–c).

**Fig. 5:**
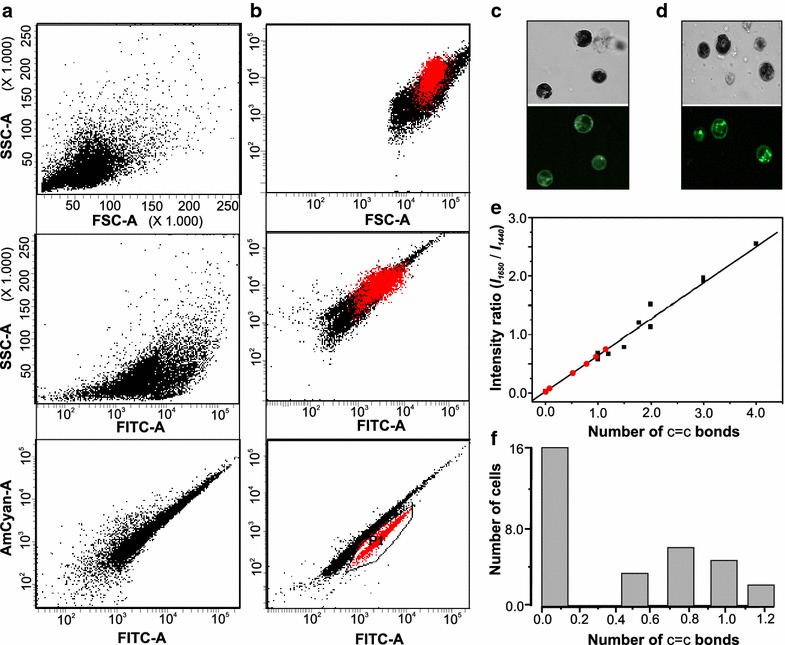
Mutagenic screen for increased lipid production. **a**, **b** *Dot plot* relating cell size (FSC-A) with the inner cell complexity (SSC-A) of cells and SSC-A with emission intensity of BODIPY in the green fluorescence bandwidth range (FITC-A and AmCyan-A) for CC-503 (**a**) and cells selected during the second round of screen (**b**). Bright field and fluorescence microscope images of cells stained with BODIPY for CC-503 wild-type (**c**) and UV-treated cells and selected during the second round of screen (**d**). Raman analysis of screened population (**e**, **f**). Raman analysis of a sample of 30 different mutants. **e** The results of ratiometric characterization for 30 different cells on calibration plot (*red squares*, some overlapping). **f** The spread of respective values

*Picochloris* sp. DN1 was found to be a picoeukaryotic alga of the prasinophyte lineage, cells ranged from 1 to 3 μm in diameter with noticeable lipid bodies observed upon staining (Fig. 4c). *Dunaliella* sp. *DN1* shared several characteristics with other Dunaliella species such as high-salt tolerance, swimming gametes, and cellular morphology (Fig. 4c). RSSF, isolated from brackish waters in the San Francisco Bay, was found to accumulate lipids to a fairly high extent. Lipids from RSSF appeared to leave the intercellular space quite easily and were often found in the extracellular space. *Chloroidium* sp. DN1, exhibited either spherical or ovoid cell morphology and ranged between 3 and 7 μm in diameter (Fig. 4c). A high degree of lipid accumulation was observed in several of the strains although no special treatments such as nitrogen-free media inoculation or UV mutagenesis was applied. To visualize the distribution of major cellular components, optical micrograph and Raman hyperspectral image of the reference *C. reinhardtii* (CC-503) microalgae is shown in Fig. 4d. Raman image of proteins (1003 cm^−1^), carotenoid components (1520 cm^−1^) and lipid bodies (1445 cm^−1^) were also constructed (Fig. 4d-3–d-5) [28]. Protein and carotenoid components are locally different than lipid bodies. The combination of these cellular components displayed in Fig. 4d-6 showed that protein and carotenes components were more dominating than the lipid bodies in the sample. This Raman hyperspectral imaging allows the localization of intact lipids stored in control microalgae sample without labeling or extraction.

### Lipid content analyses of environmental isolates

Typical aquatic and soil/fresh water microalgae spectra recorded using 532 nm excitation after photobleaching are shown in Fig. 4e, f. In both spectra, significant lipid signature (i.e., the presence of 1440 and 1650 cm^−1^ peaks) along with strong multiple lipid and carbohydrate peaks centered at 2800–3000 cm^−1^ were observed. Similar to standard fatty acids, spectra recorded for algal lipids with 532 nm excitation were dominated by 2800–3000 cm^−1^ peaks. Both fresh water and marine algal lipids also demonstrated smaller peaks around 1008 and 1520 cm^−1^, revealing carotenoid contributions. This is in agreement with previous investigations reported by Wu et al. [38], Wood et al. [39] and Heraud et al. [26, 40]. In our experiments, appearance of carotenoid bands in the Raman spectra were minimized using our refined Raman workflow, which offers the proper focusing and precise positioning of spectral acquisition locations after controlled photobleach. The algal isolates differ greatly in terms of the cell characteristics such as cell size as well as the pigments profile and the resulting fluorescence background. Yet, the ratiometric Raman method can remarkably differentiate between these diverse lipids from unrelated strains with diverse fluorescent backgrounds. The complete set of intensity ratios, number of C=C and N_C=C_/N_CH2_ for various algal lipids were evaluated. The latter showed the highest values for RSSF (lineage not determined) and *Chlamydomonas* sp. HC1 compared to other algal isolates (Table 1).

**Table 1:** Comparison of algal isolates in terms of the intensity ratios of 1650 and 1440 rel cm^−1^ spectral peaks

Microalgae	Intensity ratios	Measured C=C bonds	Measured N_C=C_/N_CH2_
*Chloroidium* sp. DN1	0.55	0.86	0.08
*Dunaliella* sp. DN1	0.57	0.89	0.08
MG8, unknown lineage	0.48	0.74	0.07
*Picochloris* sp. DN1	0.75	1.17	0.11
RSSF, unknown lineage	1.07	1.70	0.15
*Chlamydomonas* sp. KSA1	0.79	1.30	0.11
*Chlamydomonas reinhardtii* 503	0.52	0.80	0.07
*Chlamydomonas* sp. HC1	1.04	1.66	0.15

### Mutagenesic screen for increased lipid content and single cell lipid analyses

We subjected *C. reinhardtii* cells to two rounds of UV-irradiation and isolated cells with increased lipid production using FACS based on fluorescence of BODIPY 505/515 (Fig. 5a, b). BODIPY505/515 has a high oil/water partition coefficient, which allows it to easily cross cell and organelle membranes and distinctively labels the lipid components of live cells without killing the cells [41, 42]. The sorting of the stained cell populations by FACS allowed us to collect sub-populations enriched for lipid production. This technique has been used to select for mutations in several pathways unrelated to starch metabolism [43, 44].

In FACS experiments, measured forward scatter (FSC), is proportional to cell size or cell volume, while side scatter (SSC) is to the complexity of the cell. SSC intensity is affected by cell morphology, and especially by intracellular structures that are determined by chemical composition (e.g., starch, lipid content). A cell with higher level of cytoplasmic granularity, will results in higher SSC intensity [45]. The highest emission intensity of BODIPY is in the green fluorescence bandwidth range (FITC-A and AmCyan-A). Higher fluorescence emission in the FITC-A and AmCyan-A indicates high emission intensity of BODIPY as a result of its binding to neutral lipids in the cell. Hence, cell complexity (SSC) was subsequently plotted against cell size (FSC) and the fluorescence emission in the FITC-A channel. Two different cell populations were observed, in AmCyan-A vs. FITC-A plot (Fig. 5a, b). Cells forming a distinct cluster, which are highlighted in red in Fig. 5b (indicated as population P1), were sorted and used for Raman analysis. In order to qualitatively confirm the increase in mutants’ lipid content, confocal microscopy was used for detecting fluorescent label in BODIPY 505/515-stained potential mutants. Lipid bodies in unmutagenized *C. reinhardtii* (CC-503) and UV-induced mutants could be visualized and observed in a confocal microscope (Fig. 5c, d). The confocal microscope images show that the mutants have more lipid bodies and higher lipid content than the parental *C. reinhardtii* (CC-503).

Cell-to-cell analysis of variation in structural features of expressed lipids was done by assaying *C. reinhardtii* (CC-503) mutants screened for increased lipid production. Confocal Raman microscopy technique permits live, single cell observations in diverse populations, which can facilitate the observation of cell-to-cell variation and screening of cell populations for variation in expression of different TAGs. This capability provides a powerful means to explore a range of phenotypes that might be expressed within a mutagenized population. We interrogated a sample of 30 cells from the round 2 mutagenized cells, as well as wild-type cells. Cell-to-cell variation in terms of N_(C=C)_ bonds and cell numbers were observed (Fig. 5e, f). In this case, three distinct sub-populations could be identified: (a) cells that produce mostly saturated fatty acid lipids, (b) cells that produce mostly monounsaturated lipids, and (c) cells that produce a mixture of the two types. In contrast, only one type of population could be identified in the wild-type population, consisting of cells that express monounsaturated lipids only (Fig. 3c). These results demonstrate the utility of the developed method for effective screening of cells within populations with diversity in their TAG content.

## Conclusions

New isolates of algae or mutants of existing strains are potential candidates for production of industrially relevant lipids [46, 47]. However, lipid profiles of algae strains tend to be highly variable with over 76 different fatty acid species documented [48]. Bio-prospecting efforts could yield hundreds of new and uncharacterized algal species with unknown lipid profiles [49] and mutagenesis procedures often produce several hundreds to thousands of algae mutants [50, 51]. Current approaches, including analysis of lipids by GC–MS, LC–MS or HPLC tend be labor-intensive and time-consuming and hence not practical for analyzing numerous strains that may result from typical bioprospecting and mutagenesis screen endeavors. Our results demonstrate the utility of confocal Raman microscopy for rapid in situ analysis of lipids produced in microalgae. We benchmarked our Raman analysis through interrogating the well-studied reference microalgae, *C. reinhardtii* (CC-503) and cross-validated the obtained measurements with LC–MS experiments. The presented workflow and obtained results demonstrate efficient analysis of microalgal lipids on a single cell basis that can be useful in screening and monitoring, as well as metabolic engineering of the microalgae for the optimal production of biofuels. From a broader perspective, the ability to monitor lipid-based cellular processes in a label-free, quantitative manner should be broadly applicable to a variety of applications in the emerging field of lipidomics.

## Methods

### Algal strains and culture conditions

The freshwater Chlorophyte *Chlamydomonas reinhardtii*, strain CC-503 cw92 mt+ was obtained as a stab culture from the Chlamydomonas Resource Center (http://chlamycollection.org), based at the University of Minnesota (USA). CC-503 was grown on tris-acetate-phosphate (TAP) agar plates until a thick green lawn was formed. Cells were grown at 25 °C and under 400 μE of illumination. Local aquatic strains from the United Arab Emirates were isolated from areas shown in Fig. 4a. Liquid samples were collected in 50 mL collection tubes, and sampling of the gulf waters was conducted at both surface and benthic zones. Seawater samples were centrifuged at 3000*g* for 10 min. The resultant pellets were resuspended in f/2 liquid media and allowed to incubate at 25 °C with 400 μE illumination for approximately 3 days. Two hundred microliters of suspensions were then plated onto f/2 agar plates and single green colonies were isolated. Isogenic cultures were then transferred to f/2 media agar plates and harvested once a thick green lawn had formed. Soil algae, *Chlamydomonas* sp. HC1, from Abu Dhabi (Mussaffah area), UAE, and strain *Chlamydomonas* sp. KSA1, New York City (Washington Square area), NY, USA, were isolated as above except that the initial centrifugation was omitted and soil samples were initially incubated in Sager-Granick liquid medium [52] and subsequently spread on TAP-agar plates.

### Fluorescent microscopy

Cells were observed with an Olympus BX53 microscope using an UPlanFL N 100×/1.30 Oil Ph 5 UIS 2 objective (Japan) using either bright field display or fluorescence. An X-cite series 120 Q fluorescent illumination source (Lumen Dynamics, Ontario, Canada) was used to visualize Nile red and BODIPY-505/515 stained cells. Lipid staining was performed as follows: 1 μL of 10 mg/mL Nile red or BODIPY-505/515 was added to 100 μl of cells (concentration of appx. 1 × 10^6^) and allowed to incubate at room temperature in the dark for 1 h. If fluorescence quenching was too rapid to allow for imaging, cells were incubated with the dye for longer periods.

### Confocal microscopy for BODIPY-lipid visualization

Cells were observed with an Olympus Fluoview 1000 confocal laser-scanning microscope (Tokyo, Japan) equipped with 488 nm laser to visualize BODIPY 505/515 stained cells. A photo-multiplier tubes (PMT) detector was used to collect the light and the spectral filter was set from 520 to 580 nm. BODIPY staining was done as described above.

### Sequencing and phylogenetic tree reconstruction

Sequence data was obtained for the algal species using the Ion Torrent PGM (for Chlamydomonas species) and Illumina HiSeq 2500 platforms. Genomic DNA was isolated from the isolated species using Plant DNA Maxi kit (Qiagen) according to the manufacturer’s specifications. Libraries were prepared using the standard kits (Ion Express, Life Technologies) for Ion Torrent sequencing, and TrueSeq (Illumina) for HiSeq 2500 platform, according to the sequencing platform used.

The majority of sequence analysis was done using the CLC Genomics Workbench (CLC Bio, Qiagen). Fastq files of paired-end reads from the Ilumina flow cell lane were imported into CLC, followed by trimming of lower quality bases from reads (cutoff = 0.25). Trimmed reads were used for assembly. Assembly parameters were set as default except for the following: Reads were allowed to be re-mapped back to contigs, k-mer length was changed to a static 45 bp, and contigs less than 1000 bp in length were not allowed. Ribulose-1,5-bisphosphate carboxylase/oxygenase large subunit (RbcL) sequences were identified by a BLAST search of the sequenced genomes with reference sequences obtained from NCBI. Phylogenetic trees were reconstructed using a maximum-likelihood method using a Jukes-Cantor model with 1000 bootstrap replicates. The RbcL sequences used for phylogenetic analyses were deposited into Genbank under the accession numbers KP202851 (*Chloroidium* sp. DN1), KP202853 (*Dunaliella* sp. DN1), KP202854, (*Picochloris* sp. DN1), KP202855 (*Chlamydomonas* sp. HC1), and KP202852 (*Chlamydomonas* sp. KSA1).

### UV mutagenesis and FACS screen for increased lipid productivity

*C. reinhardtii* CC-503 cells were grown using TAP liquid media. 325 million cells per milliliter were transferred to TAP agar plates exposed to ultraviolet (UV) light at a distance of 30 cm (253.7 nm, 100 μW/cm^2^, 60 Hz, NuAire safety cabinets Class II Type A2 NU-425-400, http://www.nuaire.com) for 2 min under sterile conditions modified from Luck et al. [53]. The plates were subsequently kept in dark for 1 day to prevent photo reactivation. The plates were then allowed to grow under light for approximately a week [54] The resulting colonies were suspended in TAP liquid media and stained by BODIPY 505/515 for cells containing neutral lipids, which were then sorted using BD FACS Aria III instrument. The sorted cells were grown in liquid TAP media until they reached the same initial concentration, a second round of mutagenesis and sorting was done subsequently. The sorted cells from the second round of mutagenesis and sorting were used for the Raman analysis.

### Single cell confocal Raman microscopy

Confocal Raman Microscopy experiments were carried out using a combined Confocal Raman Imaging System, alpha300 RA WiTec GmbH, Germany. We used a 50× air objective with a numerical aperture of 0.8 to collect the scattered light. Accordingly, the laser spot sizes for the green (*λ* = 532 nm) and the NIR lasers (*λ* = 532 nm) are 0.811 and 1.197 µm, respectively. Theoretical (diffraction limited) lateral and axial resolutions are 0.279 and 0.971 µm, respectively, for the green (*λ* = 532 nm) laser. Corresponding values for the NIR (*λ* = 785 nm) laser are 0.412 and 1.433 µm, respectively. The sample was scanned using a piezoelectric scan table consisting of a 3-axis stage with integrated capacitive position feedback sensor to scan the sample for all modes of operation. Spectra were recorded with 600 groves/mm grating in the range of 0–3400 cm^−1^ with a spectral resolution of 3 cm^−1^. WiTec control software 16.0.3.3 version (WiTec Company, Germany) was used to record single spectra as well as spectral images.

Algal cells were washed three times with de-ionized water using centrifugation–redispersion cycles. Washed micro-algal cells were collected and transferred to clean quartz slides for Raman characterization. Fatty acid standards were analyzed by placing small droplets (for liquid samples) or specks (for solid samples) on clean quartz slides.

Characterization of algae lipids were carried out using, two lasers, 532 and 785 nm, respectively. Laser power (8 mW@532 nm and 75 mW@785 nm) and exposure times were optimally chosen through a series of control experiments to ensure that there is no laser-induced damage (i.e., formation of amorphous carbonaceous chars). Influence of fluorescence in the recorded spectrum for the 532 nm laser was eliminated by bleaching the samples for an optimized duration (30–75 s). Detailed procedure of Raman spectral acquisition is explained in Additional file 1.

For fatty acid standards and algal lipids, we used accumulation times ranging from 1 to 4 s and number of accumulations ranging from 10 to 25, respectively, to obtain signals with good signal to noise ratio. All points plotted on the calibration plots were obtained under the same acquisition conditions. The spectral and image datasets were post processed by using WiTec project plus software (WiTec Company Germany). Background resulting from cell autofluorescence, water Raman peaks, and instrumental noise was removed by using a moving average background subtraction. The filter parameters were maintained constant for both the standard lipids and the algal samples. The implementation of uniform constant filters had helped to retain the same level of residual peak distortion in all the samples. All the spectra (i.e., algal and standard lipids) presented in this paper were background corrected using the above described procedure. Lorentz multi curve fitting ≥99.9 % in area modules were used to de-convolute the measured spectra at various peak positions, namely, 1440 cm^−1^ peak (–CH_2_ stretching), 1650 cm^−1^ (C=C stretch), 2800 cm^−1^ (hydrocarbon peak), 3003 cm^−1^ peak (olefinic (=C–H stretch) stretching).

## Additional files

10.1186/s13068-015-0349-1 Schematic diagram of Raman imaging technique.
10.1186/s13068-015-0349-1 List of standard fatty acids used as calibration standards for ratiometric analysis of Raman spectra.
10.1186/s13068-015-0349-1 Curve fitting of Raman spectra recorded for oleic acid (A and B) using 532 nm at 1650 and 3003 cm^−1^.
10.1186/s13068-015-0349-1 illustration of intermixed standard fatty acids (oleic and palmitoleic acid) in the calibration curve with excitations at 532 and 785 nm.
10.1186/s13068-015-0349-1 Spectral band assignment identified in microalgal Raman spectra.
10.1186/s13068-015-0349-1 LC–MS analysis of CC-503 algal lipid extracts.

## Abbreviations

CARS: coherent anti-stokes Raman scattering
EPA: eicosapentaenoic acid
FACS: fluorescence activated cell sorting
FSC: forward scatter
HPLC: high performance liquid chromatography
LC-MS: liquid chromatography-mass analysis
LTRS: laser trapping Raman spectroscopy
NIR: near infra-red
TAG: triacylglycerol
SSC: side scatter
TAP: tris-acetate-phosphate
UV: ultraviolet light
