# Supplementary material for: An integrative Raman microscopy-based workflow for rapid in situ analysis of microalgal lipid bodies
Source: Biotechnol Biofuels. 2015 Oct 6;8:164. doi: 10.1186/s13068-015-0349-1 (PMC4595058; doi:10.1186/s13068-015-0349-1)
Supplement: Supplementary file 1 — 10.1186/s13068-015-0349-1 Schematic diagram of Raman imaging technique. [file 13068_2015_349_MOESM1_ESM.pdf]

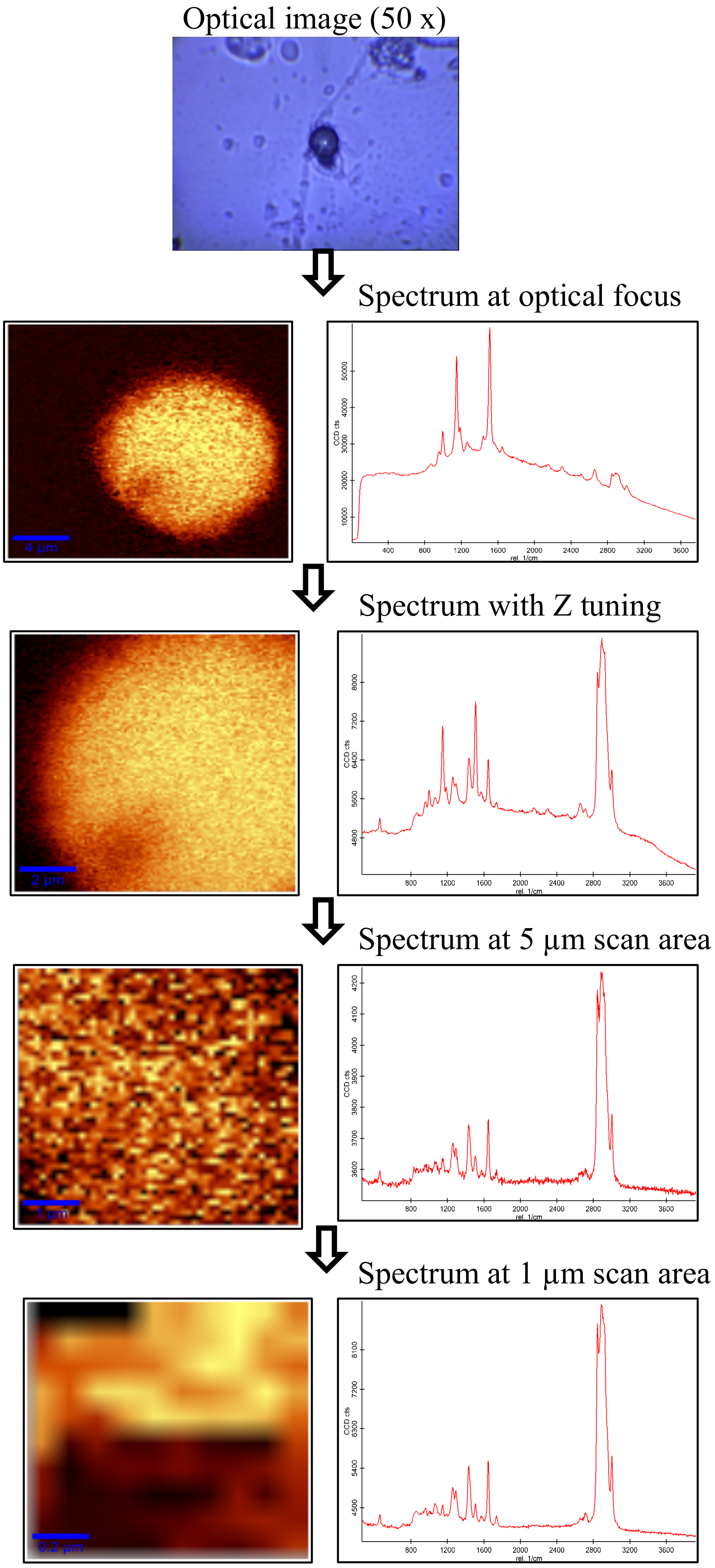

Additional file 1: Schematic diagram of Raman imaging technique. Confocal Raman microscopy is used to perform hyperspectral imaging in order to locate lipid-rich regions within microalgal cells with a high spatial resolution. Integrated peak intensity of the most intense Raman band in the lipid fingerprint region (i.e., 2800-3000 rel cm^{-1} when using the green laser for excitation and 1440 rel cm^{-1} when using the NIR laser for excitation) is used as signature to locate lipid rich regions within the cells. Imaging is initially performed over the entire cellular region and the scan area is gradually decreased to reduce the contribution of interfering, non-lipid peaks.
