# Supplementary material for: An integrative Raman microscopy-based workflow for rapid in situ analysis of microalgal lipid bodies
Source: Biotechnol Biofuels. 2015 Oct 6;8:164. doi: 10.1186/s13068-015-0349-1 (PMC4595058; doi:10.1186/s13068-015-0349-1)
Supplement: Supplementary file 2 — 10.1186/s13068-015-0349-1 List of standard fatty acids used as calibration standards for ratiometric analysis of Raman spectra. [file 13068_2015_349_MOESM2_ESM.pdf]

Additional file 2: List of standard fatty acids used as calibration standards for ratiometric analysis of Raman spectra.

Sample	Mol. Wt.	C-atoms	C=C bonds	$N_{C=C}$ /N_{CH2}	Lipid number	T_m (°C)
**Myristic acid**	228.37	14	0	0	14:0	54
**Palmitic acid**	256.42	16	0	0	16:0	63
**Lignoceric acid**	368.63	24	0	0	24:0	84
Myristoleic acid	226.36	14	1	0.11	14:1	- 4
**Palmitoleic acid**	245.41	16	1	0.08	16:1	-0.1
**Oleic acid**	282.46	18	1	0.07	18:1	13
**cis-11,14 Eicosadienoic acid**	308.50	20	2	0.13	20:2	24
**Linoleic acid**	280.45	18	2	0.15	18:2	-5
**cis-8,11,14-Eicosatrienoic acid**	306.48	20	3	0.25	20:3	NA
**Linolenic acid**	278.43	18	3	0.30	18:3	-9.5
**Arachidonic acid**	304.50	20	4	0.36	20:4	-49
