# Supplementary material for: An integrative Raman microscopy-based workflow for rapid in situ analysis of microalgal lipid bodies
Source: Biotechnol Biofuels. 2015 Oct 6;8:164. doi: 10.1186/s13068-015-0349-1 (PMC4595058; doi:10.1186/s13068-015-0349-1)
Supplement: Supplementary file 3 — 10.1186/s13068-015-0349-1 Curve fitting of Raman spectra recorded for oleic acid (A and B) using 532 nm at 1650 and 3003 cm−1. [file 13068_2015_349_MOESM3_ESM.pdf]

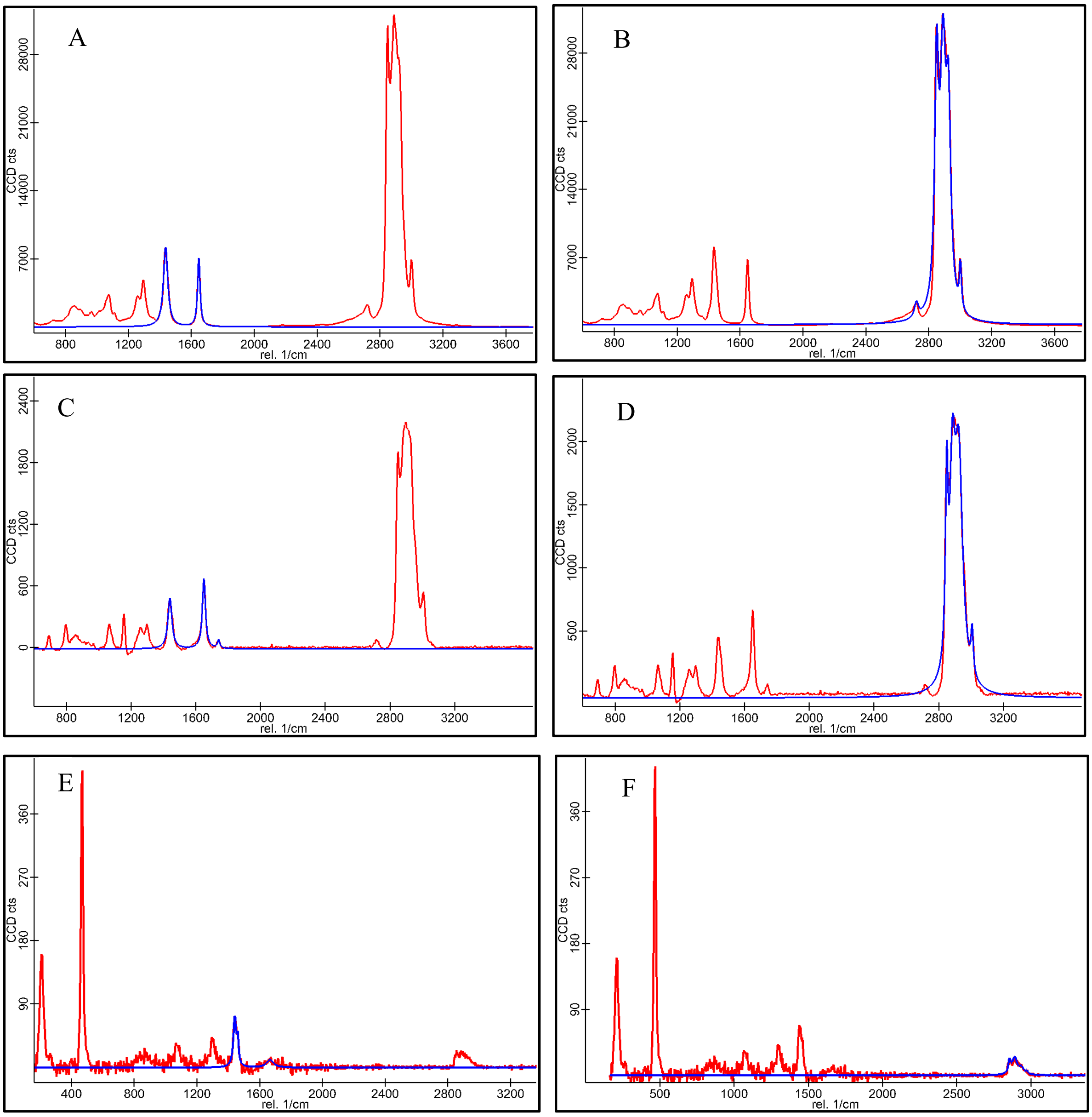


**Additional file 3:** Curve fitting of Raman spectra recorded for oleic acid (a and b) using 532 nm at 1650 $cm^{-1}$ and 3003 $cm^{-1}$, respectively. Spectra c to f were recorded for *C. reinhardtii* (CC-503) microalgae using 532 nm (c and d) and 785 nm ( e and f) excitations, with curve fitting at 1650 $cm^{-1}$ and 3003 $cm^{-1}$, respectively. Red curves represent the background corrected Raman spectra whereas blue curves are results of curve-fitting respective band envelopes using combinations of Lorentzian peaks.

**Curve fitting information of 1650 $cm^{-1}$ with 3003 $cm^{-1}$ bands:**

The intensity ratio values ($I_{1650}$ / $I_{1440}$) and ($I_{2900}$/$I_{1440}$) evaluated using Lorentzian curve fitting 1650 $cm^{-1}$ and the additional olefinic (=C–H stretch) peak located at 3003 $cm^{-1}$ with respect to 1440 peak for a pure fatty acid standard (oleic acid) and microalgae samples. As a proof of validation, we demonstrate the curve fitting and evaluated intensity ratios (shown in the table below) for oleic acid and the control, *C. reinhardtii* (CC-503) microalgae sample, using 532

and 785 nm excitations.

While comparing the intensity ratios 1650/1440, a significant overlap of $-CH_2$ stretch located ~2900 $cm^{-1}$ can be observed, which in turn results a decrease in carbonyl peak intensity ratio 2900/1440 but found to be satisfactory under these constraints.

| **Sample details** | **Excitation source** | $I_{1650}/I_{1440}$ | $I_{2900}/I_{1440}$ |
|---|---|---|---|
| **Oleic acid (A and B)** | 532 nm | 0.56 | 0.40 |
| ***C. reinhardtii*** **(CC-503) ( C and D)** | 532 nm | 0.52 | 0.38 |
| ***C. reinhardtii*** **(CC-503) ( E and F*)*** | 785 nm | 0.30 | 0.18 |
