# Supplementary material for: An integrative Raman microscopy-based workflow for rapid in situ analysis of microalgal lipid bodies
Source: Biotechnol Biofuels. 2015 Oct 6;8:164. doi: 10.1186/s13068-015-0349-1 (PMC4595058; doi:10.1186/s13068-015-0349-1)
Supplement: Supplementary file 4 — 10.1186/s13068-015-0349-1 illustration of intermixed standard fatty acids (oleic and palmitoleic acid) in the calibration curve with excitations at 532 and 785 nm. [file 13068_2015_349_MOESM4_ESM.pdf]

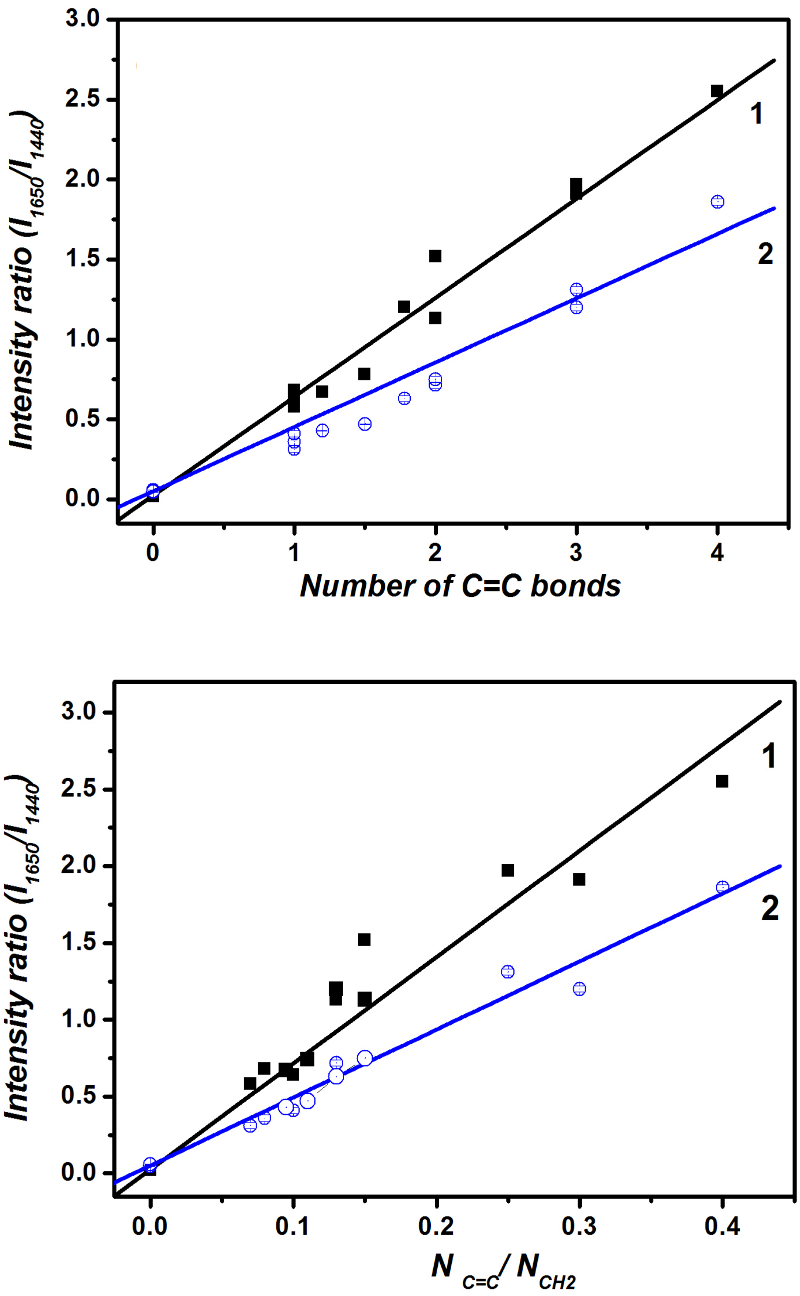

Additional file 4: illustration of intermixed standard fatty acids (oleic and palmitoleic acid) in the calibration curve with excitation of (1) 532 nm and (2) 785 nm.
