# Supplementary material for: An integrative Raman microscopy-based workflow for rapid in situ analysis of microalgal lipid bodies
Source: Biotechnol Biofuels. 2015 Oct 6;8:164. doi: 10.1186/s13068-015-0349-1 (PMC4595058; doi:10.1186/s13068-015-0349-1)
Supplement: Supplementary file 5 — 10.1186/s13068-015-0349-1 Spectral band assignment identified in microalgal Raman spectra. [file 13068_2015_349_MOESM5_ESM.pdf]

Additional file 5: Spectral band assignment identified in microalgal Raman spectra.

Wave number (rel. cm)$^{-1}$	Band assignment
744	δH-C-O, δN-C-C chla
915	δN-C-C, δC-C-C chla
988	δCH_3 chla
1004-1008	Carotene C-H bending, ρ CH3, υCC β- carotene
1056, 1116	C-C *trans* and *gauche* stretching,
1060	C-C skeleton stretching
1075	C-C *gauche* stretching
1082	C-O-H bending
1085	C-C skeleton stretching
1120	C-O-H deformation, C-O stretching
1125	C-C skeleton stretching
1157, 1160	β- carotene, carotene C-H stretching,
1187	δCH, υC-N chla (1186 cm^{-1}) δCH β- carotene (1191 cm^{-1})
1230	δCH, υCC υC-N chla
1260	*cis* double bond =C-H bending
1265	δCH_3 β- carotene and / or amide III of protein
1289	δCH_3, δCH, υC-N chla
1300	CH_2 twisting
1308	δCH_3, δCH chla
1325	υC–N, δCH chla
1340	CH_2 deformation, cholorophyll C-N stretching
1348	δCH_3, δCH, υC–N chla
1389	δCH_3 , δCH, υC-N chla, δCH_3 β- carotene
1438	υCC, , δCH_3 chla, , δCH_3 chla β- carotene
1440	CH_2 bending
1495	υCC, δCH_3 chla
1500-1600	β- carotene (C=C stretching)
1525	$Y_{(C=C)}$ β-carotene (1524 cm^{-1}) υ_{CC} chla (1535 cm^{-1})
1605	υ_{CC} chla
1650	*cis* C=C stretching
1670	υC=O chla and /or amide I of protein
1736	C=O stretches , ester stretching
2800-2930	CH_2, CH_3 symmetric and asymmetric stretching
3023	=C-H stretching
