# Supplementary material for: An integrative Raman microscopy-based workflow for rapid in situ analysis of microalgal lipid bodies
Source: Biotechnol Biofuels. 2015 Oct 6;8:164. doi: 10.1186/s13068-015-0349-1 (PMC4595058; doi:10.1186/s13068-015-0349-1)
Supplement: Supplementary file 6 — 10.1186/s13068-015-0349-1 LC–MS analysis of CC-503 algal lipid extracts. [file 13068_2015_349_MOESM6_ESM.pdf]

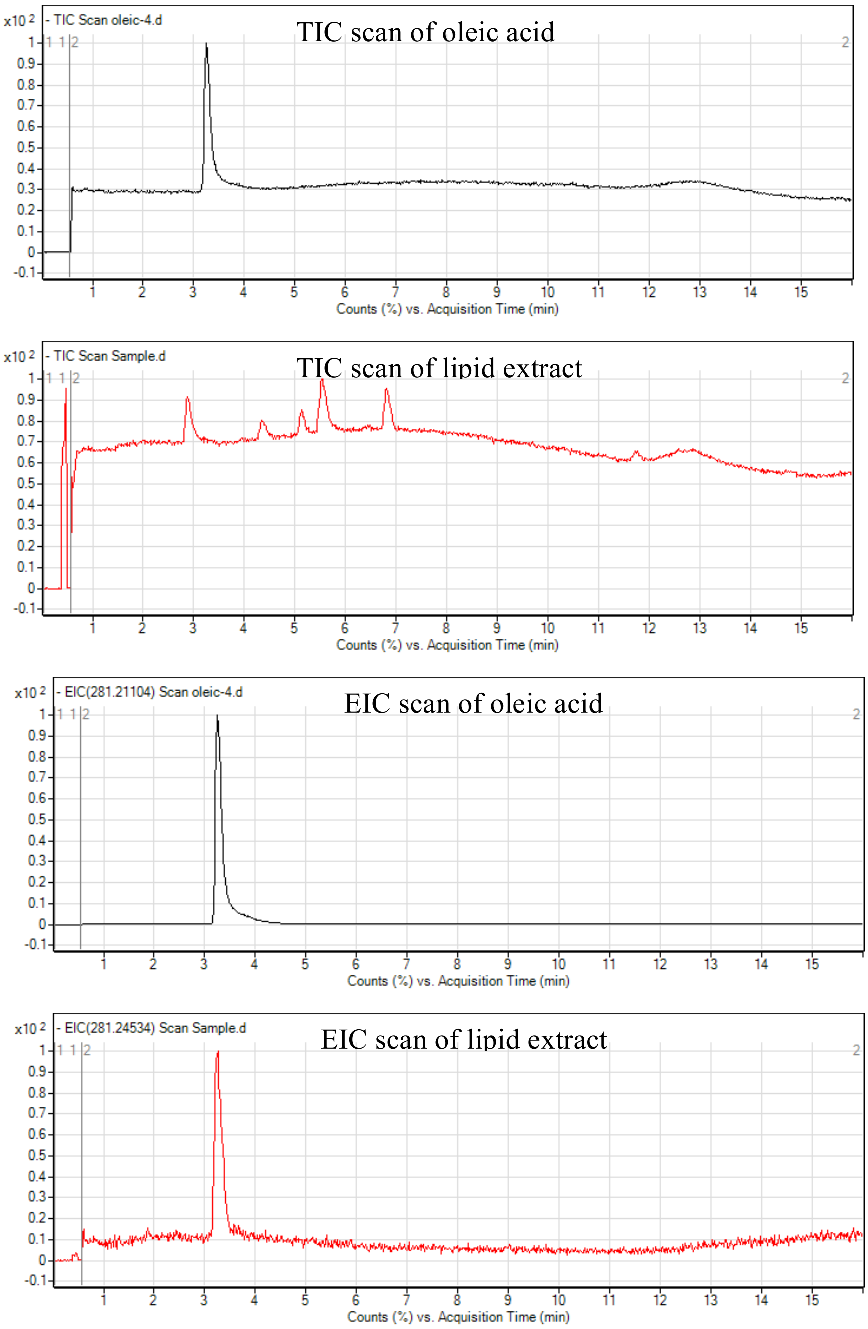

Additional file 6:

LC-MS analysis of CC-503 algal lipid extracts was carried out for independent verification of the results obtained from ratiometric Raman analysis. Figure above shows the TICs (total ion count) and EICs (extracted ion chromatograph) of the standard oleic acid solution and the lipid extract after hydrolysis.
